# Fluorescein-guided surgery for high-grade glioma resection: a five-year-long retrospective study at our institute

**DOI:** 10.3389/fonc.2023.1191470

**Published:** 2023-06-02

**Authors:** Chen Xi, Sun Jinli, Mao Jianyao, Chen Yan, Li Huijuan, Shi Zhongjie, Li Zhangyu, Zhou Liwei, Li Yukui, Chen Sifang, Tan Guowei

**Affiliations:** ^1^ Department of Neurosurgery, The First Affiliated Hospital of Xiamen University, Xiamen, Fujian, China; ^2^ Department of Reproduction, The First Affiliated Hospital of Xiamen University, Xiamen, Fujian, China; ^3^ Department of Orthopedic Sports Medicine, The First Affiliated Hospital of Xiamen University, Fujian, China; ^4^ Department of Trauma Center and Acute Abdomen Surgery, The First Affiliated Hospital of Xiamen University, Fujian, China

**Keywords:** sodium fluorescein, fluorescein-guided surgery, high-grade glioma, gross-total resection, extent of resection

## Abstract

**Objective:**

This study investigates the extent of resection, duration of surgery, intraoperative blood loss, and postoperative complications in patients with high-grade glioma who received surgery with or without sodium fluorescein guidance.

**Methods:**

A single-center retrospective cohort study was conducted on 112 patients who visited our department and underwent surgery between July 2017 and June 2022, with 61 in the fluorescein group and 51 in the non-fluorescein group. Baseline characteristics, intraoperative blood loss, surgery duration, resection extent, and postoperative complications were documented.

**Results:**

The duration of surgery was significantly shorter in the fluorescein group than in the non-fluorescein group (P = 0.022), especially in patients with tumors in the occipital lobes (P = 0.013). More critically, the gross total resection (GTR) rate was significantly higher in the fluorescein group than in the non-fluorescein group (45.9% vs. 19.6%, P = 0.003). The postoperative residual tumor volume (PRTV) was also significantly lower in the fluorescein group than in the non-fluorescein group (0.40 [0.12-7.11] cm^3^ vs. 4.76 [0.44-11.00] cm^3^, P = 0.020). Particularly in patients with tumors located in the temporal and occipital lobes (temporal, GTR 47.1% vs. 8.3%, P = 0.026; PRTV 0.23 [0.12-8.97] cm^3^ vs. 8.35 [4.05-20.59] cm^3^, P = 0.027; occipital, GTR 75.0% vs. 0.0%, P = 0.005; PRTV 0.15 [0.13-1.50] cm^3^ vs. 6.58 [3.70-18.79] cm^3^, P = 0.005). However, the two groups had no significant difference in intraoperative blood loss (P = 0.407) or postoperative complications (P = 0.481).

**Conclusions:**

Fluorescein-guided resection of high-grade gliomas using a special operating microscope is a feasible, safe, and convenient technique that significantly improves GTR rates and reduces postoperative residual tumor volume when compared to conventional white light surgery without fluorescein guidance. This technique is particularly advantageous for patients with tumors located in non-verbal, sensory, motor, and cognitive areas such as the temporal and occipital lobes, and does not increase the incidence of postoperative complications.

## 1 Introduction

Gliomas arise from glial cells in the brain and are the most common primary intracranial tumors, accounting for 80% of all primary malignant tumors of the central nervous system. The annual incidence of glioma in China is (3-6.4)/100,000, with 30,000 deaths annually. Gliomas are generally classified into high-grade glioma (HGG) (grade 3/4) and low-grade glioma (grade 1/2) according to their pathology (1, 2). Treatment of HGGs is based on surgical resection combined with radiotherapy and chemotherapy. Surgery can relieve clinical symptoms, prolong survival, and provide sufficient tumor samples for definitive pathological diagnosis and molecular genetic testing (3). Safe maximal surgical resection (resection of all contrast-enhancing tissue and supramaximal resection if possible) is the principle of management for HGGs (4, 5). However, because of the biological characteristics of HGGs with indistinct boundaries and infiltrative growth, it is difficult to identify the tumor boundaries intraoperatively and achieve complete histological resection in such cases (6). Recently, the use of new surgical techniques such as fluorescence-guided surgery (FGS), neuronavigation, intraoperative neurophysiological monitoring, and real-time intraoperative magnetic resonance imaging (MRI) have maximized the safe resection of HGGs (7). Recently, advanced confocal laser technology, such as confocal endoscopy, provides real-time intraoperative histological images. Therefore, this improves visualization in HGGs by clearly identifying brain tumors, microvessels, and tumor margins (8, 9).

Sodium fluorescein (SF), with the molecular formula C20H10Na2O5, is a small molecule dye with fluorescence properties (relative molecular mass: 376). This dye is a non-targeting tracer that does not cross the blood-brain barrier (BBB) and cannot enter or specifically bind to tumor cells. However, HGGs exhibit diffuse infiltrative growth and invade the surrounding blood vessels, causing damage to the endothelial ultrastructure and altering the permeability of the vessel wall; this disrupts the BBB and allows SF to enter and accumulate in the tumor tissue. Yellow-green fluorescence can be observed in tumor tissue at a wavelength of 560 nm using a fluorescent light source, which can be used to map the tumor boundary. The degree of BBB damage increases with the glioma grade, while the effect of SF becomes more pronounced (7, 10, 11). In 2011, Acerbi et al. initiated a prospective phase II clinical trial (FLUOGLIO) to evaluate the safety and efficacy of FGS (5-10 mg/kg) of HGGs using a Pentero microscope (Carl Zeiss, Germany) equipped with a special YELLOW 560 filter. The results showed no adverse effects associated with using SF, and gross total resection (GTR) of contrast-enhanced tumors was achieved in 75% of the 12 World Health Organization (WHO) grade 4 gliomas (12). Since then, many studies have confirmed the safety and efficacy of fluorescein-guided resection of HGGs (13–16). Although 5-aminolevulinic acid (5-ALA) remains the only drug approved for FGS in HGG resection, recent studies have shown that the advantages of SF over 5-ALA include a wide range of indications, low cost, non-toxicity, and ease of intraoperative administration. It may be a reasonable and feasible alternative to 5-ALA for fluorescence-guided HGG surgery (4, 17). Furthermore, SF may increase the GTR rate in recurrent glioblastoma with minimal risk and clear margins (18). and patients with primary central nervous system lymphoma, brain abscesses or cerebral metastases may also benefit from using SF and the dedicated 560 nm YELLOW filter during craniotomy (19–22). In non-enhancing low-grade gliomas, SF can identify locations of vascular dysregulation lesions associated with high-grade features (23). In 2018, our neurosurgery department gradually introduced SF-guided surgery for HGGs by acquiring an OPMI Pentero 900 microscope (Carl Zeiss, Germany) with a special YELLOW 560 filter.

However, few studies have comprehensively compared fluorescein-guided and non-fluorescein-guided HGG resection. Therefore, in this study, we retrospectively analyzed data from patients who underwent HGG surgical resection with or without SF guidance in the last five years and performed statistical analysis of surgical outcomes and adverse events. This single-center retrospective cohort study investigated the extent of resection (EOR), duration of surgery, intraoperative blood loss, and postoperative complications in patients with HGG who received surgery with or without SF guidance. We present the following article based on the STROBE checklist (available at https://www.strobe-statement.org/checklists).

## 2 Materials and methods

### 2.1 Patients

The medical records of 112 patients who visited our neurosurgery department between July 2017 and June 2022 and received surgery were retrospectively analyzed. Inclusion criteria: (1) newly diagnosed or recurrent HGG suitable for surgical resection; (2) no contraindications to surgery, and the patient and family consented to surgery; and (3) pathological examination showed WHO grade 3/4 glioma. The exclusion criteria were: (1) cardiopulmonary, hepatic, or renal dysfunction, coagulopathy, or other underlying systemic disease contraindicating surgery; (2) history of active malignancy elsewhere; (3) medical reasons precluding MRI enhancement; (4) multicentric lesion or biopsy only; (5) recurrent HGG; and (6) presence of a large non-contrast-enhancing area suggestive of low-grade glioma with malignant transformation. This study protocol was formulated according to the World Medical Association’s Declaration of Helsinki requirements. The Ethics Committee of the First Affiliated Hospital of Xiamen University [No. 2023KYEC (007)] approved it.

### 2.2 Clinical and radiological assessment

The clinical evaluation included postoperative assessments, with the time points being 7 days before surgery, 24-72 h after surgery, and 4 weeks after surgery. Basic information such as age, gender, weight, height, improvement of main clinical symptoms after surgery, and changes in laboratory test data was recordedwere recorded for all patients. Immediate and postoperative responses to the use of SF were assessed for objective circumstances. Subjective outcomes could not be assessed because of the limitations of general anesthesia.

In all cases, preoperative MRI was performed 7 days before surgery, and postoperative MRI was performed 24-72 h after surgery. The primary endpoint of the study was the difference between the two groups in terms of EOR, as determined by the Response Assessment in Neuro-Oncology criteria (24), where measurable residuals were defined as lesions with a maximum diameter and a second vertical measurement of ≥ 10 mm. Patients classified as having “no residual tumor” or “unmeasurable residual” were pooled into the gross total resection (GTR) group. In contrast, patients with “measurable residual lesions’ were considered the partial resection (PR) group (4). Preoperative tumor volume and postoperative residual tumor volume (PRTV) were calculated using open-source software (Osirix for Macintosh, available at https://www.osirix-viewer.com) (25). Secondary endpoints included the duration of surgery, intraoperative blood loss, and incidence of postoperative complications.

### 2.3 Surgical protocol

Before anesthesia, 50 mg of the agent (0.5 mL of a 100 mg/mL stock solution, Alcon Laboratories, USA) was administered to patients receiving SF injections. The dye was diluted to 5 mL with 0.9% NS and injected intravenously into the patient. When no allergic reaction was observed within 20 min, the patient received intravenously 3-5 mg/kg of SF. The subdural manipulation was performed at least 90 min later to allow sufficient time for SF to accumulate in the brain tumor tissue.

**Figure 1:**
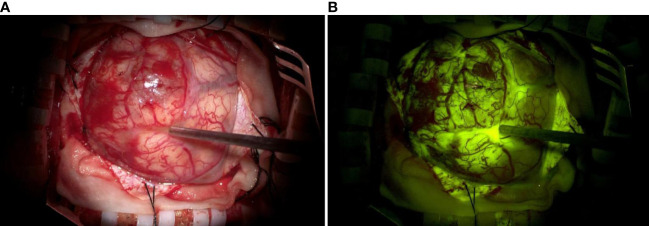
**(A)** Representative image of a glioma visualized with a conventional white light microscope without fluorescent labeling. **(B)** The same glioma was visualized with fluorescent labeling using a microscope with a YELLOW 560 nm filter. The tumor margins are visible in **(B)**.

**Figure 2:**
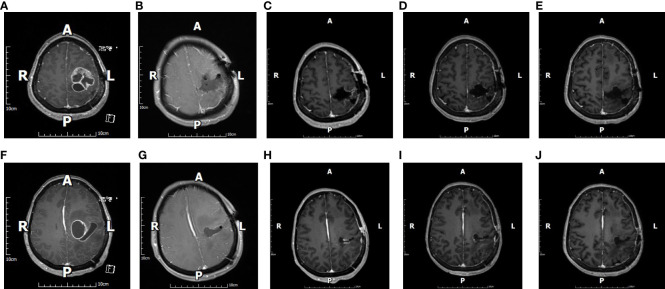
The figure shows images of a 45-year-old female patient with glioblastoma. Preoperative cranial magnetic resonance imaging (MRI)-enhanced images **(A, F)** suggest that the tumor was located in the left parietal lobe, and images taken at the same sections 48 h after surgery **(B, G)** show gross total resection (GTR). Cranial MRI-enhanced images were reviewed in the same sections 1 month after surgery **(C, H)**, 2 months after surgery **(D, I)**, and 6 months after surgery **(E, J)**. All images were suggestive of GTR.

The appropriate surgical approach was selected based on preoperative imaging, and the cortical incision, flap size, and surgical approach were designed according to neuronavigation. Craniotomy was performed, and the dura was incised. The surgical site in the fluorescein group was visualized in the yellow 560 fluorescence mode to identify the tumor location and boundaries (**Figure 1**). The estimated EOR was determined according to the stained margins to ensure GTR or PR of the tumor tissue. Patients in the non-fluorescence-guided surgery (NFGS) group underwent tumor resection under conventional white light microscopy with the assistance of neuronavigation. The dura was tightly sutured, the bone flap was repositioned, and the scalp was finally sutured after complete hemostasis of the surgical area. Intraoperative blood loss and the duration of surgery were documented. Patients were admitted to the neurological intensive care unit after surgery and received postoperative MRI enhancement within 72 h to assess EOR (**Figure 2**).

### 2.4 Pathological classification

Each patient’s pathology of tumor tissue was classified according to the WHO fifth edition classification of central nervous system tumors, WHO CNS5, 2021 (4).

### 2.5 Postoperative complication evaluation

Postoperative complications were graded according to a revised Clavien-Dindo classification of surgical complications to assess the severity at 4 weeks postoperatively. Five levels were included, i.e., I: any deviation from the normal required no treatment, II: required medication, III: required intervention, IV: required intensive care, and V: death (26–28).

### 2.6 Statistical analyses

SPSS software (version 26.0) was used for the statistical analysis of the data. Two independent samples t-tests were used to compare data that showed a normal distribution. Enumeration data were expressed as percentages or proportions, and comparisons between groups were made using the Mann–Whitney U test of two independent samples in a non-parametric test. Interaction and stratified analyses were conducted according to the adoption of SF-guided resection, EOR and tumor locations. Statistical significance was set at P < 0.05.

## 3 Results

The study included 112 cases; 61 cases were included in the fluorescein group (34/27 male/female, mean age 53.20 ± 13.38 years); 51 cases were included in the non-fluorescein group (33/18 male/female, mean age 51.63 ± 16.89 years). All patients received surgery and completed cranial MRI enhancement within 72 h of surgery to assess EOR. There were no statistically significant differences in the baseline characteristics between the two groups of patients (**Table 1**).

**Table 1:** Baseline characteristics of patients with different tumor locations between Fluorescein/Non-fluorescein groups.

Characteristics		Frontal			Temporal			Parietal			Occipital			Deep supratentorial (Insular lobe, corpus callosum, basal ganglia)			Total		
		FG n = 22	NFG n = 12	*P*	FG n = 17	NFG n = 12	*P*	FG n = 6	NFG n = 13	*P*	FG n = 8	NFG n = 6	*P*	FG n = 8	NFG n = 8	*P*	FG n = 61	NFG n = 51	*P*
Age, year, M ± SD		52.14 (15.22)	49.83 (8.33)	0.632	54.47 (13.37)	52.75 (18.38)	0.772	47.50 (11.22)	57.31 (12.80)	0.126	56.87 (13.29)	57.00 (15.74)	0.987	54.00 (10.57)	39.38 (25.98)	0.173	53.20 (13.38)	51.63 (16.89)	0.584
Sex, n (%)				0.493			0.979			0.637			0.533			0.302			0.335
Male		12 (54.5)	8 (66.7)		10 (58.8)	7 (58.3)		4 (66.7)	10 (76.9)		4 (50.0)	2 (33.3)		4 (50.0)	6 (75.0)		34 (55.7)	33 (64.7)	
Female		10 (45.5)	4 (33.3)		7 (41.2)	5 (41.7)		2 (33.3)	3 (23.1)		4 (50.0)	4 (66.7)		4 (50.0)	2 (25.0)		27 (44.3)	18 (35.3)	
BMI, kg/m^2^, M ± SD		24.11 (8.80)	22.30 (2.23)	0.574	23.71 (3.09)	23.55 (4.49)	0.917	24.26 (3.19)	21.73 (2.61)	0.144	22.85 (2.40)	22.29 (2.71)	0.705	28.42 (12.97)	22.16 (2.84)	0.273	24.41 (7.31)	22.45 (3.02)	0.139
Pathological classification, n (%)				0.688			0.345			0.130			0.231			0.158			0.205
Glioblastoma, IDH-wide type		14 (63.6)	9 (75.0)		16 (94.1)	11 (91.7)		5 (83.3)	13 (100.0)		8 (100.0)	5 (83.3)		5 (62.5)	8 (100.0)		48 (78.7)	46 (90.2)	
Grade 3 IDH-mutant, 1p/19q co-deleted oligodendroglioma		3 (13.6)	2 (16.7)		0	1 (8.3)		1 (16.7)	0		0	1 (16.7)		2 (25.0)	0		6 (9.8)	4 (7.8)	
Grade 3 IDH-mutant astrocytoma		2 (9.1)	0		1 (5.9)	0		0	0		0	0		1 (12.5)	0		4 (6.6)	0	
Grade 4 IDH-mutant astrocytoma		3 (13.6)	1 (8.3)		0	0		0	0		0	0		0	0		3 (4.9)	1 (2.0)	

FG, fluorescein group; NFG, non-fluorescein group; M ± SD, mean ± standard deviation; BMI, body mass index.

The duration of surgery was significantly shorter in the fluorescein group than in the non-fluorescein group (P = 0.022, **Table 2**). More critically, the GTR rate was significantly higher in the fluorescein group than in the non-fluorescein group (45.9% vs. 19.6%, P = 0.003, **Table 2**). The PRTV was also significantly smaller in the fluorescein group than in the non-fluorescein group (0.40 [0.12-7.11] cm^3^ vs. 4.76 (0.44-11.00) cm^3^, P = 0.020, **Table 2**). There were no significant differences between the two groups in intraoperative blood loss (P = 0.407, **Table 2**) or the incidence of postoperative complications (P = 0.481, **Table 2**).

**Table 2:** Intra-operative and postoperative comparisons of patients with different tumor locations between the fluorescein and non-fluorescein groups.

Variables, n (%)		Frontal			Temporal			Parietal			Occipital			Deep supratentorial (Insular lobe, corpus callosum, basal ganglia)			Total		
		FG n = 22	NFG n = 12	*P*	FG n = 17	NFG n = 12	*P*	FG n = 6	NFG n = 13	*P*	FG n = 8	NFG n = 6	*P*	FG n = 8	NFG n = 8	*P*	FG n = 61	NFG n = 51	*P*
Intraoperative blood loss		464.32 (462.76)	421.25 (357.15)	0.782	414.71 (353.45)	363.75 (322.61)	0.695	816.67 (953.76)	307.69 (239.66)	0.251	469.37 (302.38)	867.50 (1084.87)	0.337	387.50 (290.01)	232.50 (196.45)	0.231	475.74 (467.25)	401.67 (470.31)	0.407
Duration of surgery		324.55 (108.65)	350.42 (86.98)	0.484	360.29 (84.60)	357.08 (85.82)	0.921	376.67 (103.86)	400.00 (94.27)	0.633	283.75 (74.25)	449.17 (138.15)	0.013	417.50 (138.62)	365.63 (154.17)	0.491	346.48 (106.50)	378.63 (108.16)	0.022
Extent of resection				0.354			0.026			0.141			0.005			0.522			0.003
Gross total resection		9 (40.9)	3 (25.0)		8 (47.1)	1 (8.3)		4 (66.7)	4 (30.8)		6 (75.0)	0 (0)		1 (12.5)	2 (25.0)		28 (45.9)	10 (19.6)	
Partial resection		13 (59.1)	9 (75.0)		9 (52.9)	11 (91.7)		2 (33.3)	9 (69.2)		2 (25.0)	6 (100.0)		7 (87.5)	6 (75.0)		33 (54.1)	41 (80.4)	
Preoperative tumor volume in cm^3^, median (IQR)		42.15 (27.63-62.35)	41.40 (28.40-58.40)	0.894	35.70 (21.65-49.45)	43.10 (23.83-66.65)	0.330	37.80 (27.85-69.00)	39.30 (18.05-58.10)	0.512	32.15 (29.48-52.43)	45.05 (25.38-87.45)	0.519	41.05 (12.68-63.65)	23.30 (17.10-35.83)	0.423	39.10 (27.80-59.70)	39.20 (22.30-58.40)	0.944
Postoperative residual tumor volume in cm^3^, median (IQR)		1.75 (0.13-14.90)	3.74 (0.17-17.71)	0.688	0.23 (0.12-8.97)	8.35 (4.05-20.59)	0.027	0.09 (0.05-3.36)	2.23 (0.11-10.13)	0.122	0.15 (0.13-1.50)	6.58 (3.70-18.79)	0.005	5.90 (0.33-12.05)	3.04 (0.14-6.79)	0.522	0.40 (0.12-7.11)	4.76 (0.44-11.00)	0.020
Postoperative Complications				0.763			0.927			0.637			0.733			0.301			0.481
Grade I		5 (22.7)	3 (25.0)		4 (23.5)	3 (25.0)		2 (33.3)	3 (23.1)		2 (25.0)	2 (33.3)		2 (25.0)	2 (25.0)		15 (24.6)	13 (25.5)	
Grade II		0 (0)	0 (0)		0 (0)	0 (0)		0 (0)	0 (0)		0 (0)	0 (0)		2 (25.0)	0 (0)		2 (3.3)	0 (0)	
Grade III		1 (4.5)	0 (0)		0 (0)	0 (0)		0 (0)	0 (0)		0 (0)	0 (0)		0 (0)	0 (0)		1 (1.6)	0 (0)	
Grade IV		1 (4.5)	0 (0)		0 (0)	0 (0)		0 (0)	0 (0)		0 (0)	0 (0)		0 (0)	0 (0)		1 (1.6)	0 (0)	
No complications		15 (68.2)	9 (75.0)		13 (76.5)	9 (75.0)		4 (66.7)	9 (75.0)		6 (75.0)	4 (66.7)		4 (50.0)	6 (75.0)		42 (68.9)	38 (74.5)	

FG, fluorescein group; NFG, non-fluorescein group.

We further classified the cases into five categories based on tumor location: frontal, temporal, parietal, occipital, and deep supratentorial (insula, corpus callosum, basal ganglia). The case distribution in the groups based on the use of fluorescein was as follows: total number in the group, number in fluorescein/non-fluorescein groups: frontal group 34, 22/12; temporal group: 29, 17/12; parietal group: 19, 6/13; occipital group: 14, 8/6; and deep supratentorial group (insula, corpus callosum, basal ganglia) 16, 8/8. There were no statistically significant differences in baseline characteristics between the fluorescein and non-fluorescein subgroups within the tumor location categories (**Table 1**). Intraoperative blood loss (frontal, P = 0.782; temporal, P = 0.695; parietal, P = 0.251; occipital, P = 0.337; deep supratentorial, P = 0.231; **Table 2**) and incidence of postoperative complications (frontal, P = 0.763; temporal, P = 0.927; parietal, P = 0.637; occipital, P = 0.733; deep supratentorial, P = 0.301; **Table 2**) were also not significantly different between the two groups. In patients with tumors in the occipital lobes, the duration of surgery was significantly shorter in the fluorescein group than in the non-fluorescein group (P = 0.013; **Table 2**). Contrarily, in patients with tumors in the frontal, temporal, parietal lobes and the deep supratentorial area, there was no significant difference between the two groups (frontal, P=0.484; temporal, P = 0.921; parietal, P = 0.633; deep supratentorial, P = 0.491; **Table 2**).

In patients with tumors located in the temporal and occipital lobes, the GTR rate was significantly higher in the fluorescein group compared to the non-fluorescein group (temporal: GTR 47.1% vs. 8.3%, P = 0.026; occipital: GTR 75.0% vs. 0.0%, P = 0.005; **Table 2**). Additionally, the PRTV was significantly smaller in the fluorescein group than in the non-fluorescein group (temporal: 0.23 [0.12-8.97] cm^3^ vs. 8.35 [4.05-20.59] cm^3^, P = 0.027; occipital: 0.15 [0.13-1.50] cm^3^ vs. 6.58 [3.70-18.79] cm^3^, P = 0.005; **Table 2**). In patients with tumors located in the frontal lobe, parietal lobe, and deep supratentorial region, no significant difference was observed between the two groups (frontal: 40.9% vs. 25.0%, P = 0.354; parietal: 66.7% vs. 30.8%, P = 0.141; deep supratentorial region: 12.5% vs. 25.0%, P = 0.522; **Table 2**). The PRTV also showed no significant statistical difference between the two groups (frontal: 1.75 [0.13-14.90] cm^3^ vs. 3.74 [0.17- 17.71] cm^3^, P = 0.688; parietal: 0.09 [0.05-3.36] cm^3^ vs. 2.23 [0.11-10.13] cm^3^, P = 0.122; deep supratentorial region: 5.90 [0.33-12.05] cm^3^ vs. 3.04 [0.14-6.79] cm^3^, P = 0.522; **Table 2**). We additionally performed binary logistic regression with EOR as the dependent variable to calculate the interaction between tumor location and adoption of SF-guided resection, and the results showed a statistically significant difference in the interaction P value (P = 0.037, **Table 3**).

**Table 3:** Interaction and stratified analyses according to the adoption of SF-guided resection, EOR and tumor locations.

Locations	FG		NFG		p Value for interaction
	GTR n=28	PR n=33	GTR n=10	PR n=41	
					0.037
Temporal & Occipital	14	11	1	17	
Frontal, Parietal & Deep supratentorial	14	22	9	24	

FG, fluorescein group; NFG, non-fluorescein group; GTR, gross-total resection; PR, partial resection.

## 4 Discussion

Sodium fluorescein (SF), a fluorescent compound discovered by Adolf von Baeyer in 1871, has typically been used in ocular retinal angiography (2 9, 29). However, as early as 1947, Moore et al. studied the role of SF in neuro-oncological procedures and examined the differences in fluorescein signal intensity between malignant and benign tissues (30). The mechanism of action of SF is thus very similar to gadolinium. Consequently, this reduces the impact of deficiencies in conventional neuronavigation, such as brain displacement or registration errors and allows neurosurgeons to visualize contrast-enhanced areas of tumors in real time (15, 29, 31–33). Some data in the current literature reveals that SF has satisfactory sensitivity and specificity on tumor tissue, reaching up to 82-94% and 90-91%, respectively (34). Additionally, in a multicenter, prospective phase II clinical trial of SF-guided resection of HGGs (FLUOGLIO) conducted by Acerbi F et al., 50 biopsies were performed at the margins of tumor tissue in 13 patients (26 biopsies of fluorescent tissue and 24 biopsies of non-fluorescent tissue), and the sensitivity and specificity of tumor identification were 80.8% and 79.1%, respectively. The positive predictive value (PPV) and negative predictive value (NPV) were 80.8% and 79.1%, respectively (35). A retrospective single-center cohort study published by Sweeney JF et al. showed that the sensitivity and specificity of SF were 62% and 100% (36). However, the sensitivity and specificity of SF on tumor tissue is affected by the conditionality of the surgical sample, especially in peri-tumor “healthy” tissue, which must also be acknowledged (35).

The potential of fluorescein-guided surgery (FGS) to significantly improve GTR rates in HGGs has been demonstrated in several studies (7, 15, 23, 37–40). Smith et al. evaluated the GTR rate of fluorescein-guided HGG surgery in a meta-analysis study. 336 patients underwent fluorescein-guided HGG resection with a GTR rate of 81% (95% confidence interval 73%-89%; p < 0.001). They further analyzed 10 case-control studies and reported a 29.5% increase in the GTR rate in the fluorescein-guided group compared to the non-fluorescein-guided group (41). Acerbi et al. analyzed data from 412 articles, 17 closely related to fluorescein-guided resection of HGGs. They reported that fluorescein-guided surgery is a safe, effective, and convenient technique for achieving high GTR rates in HGGs. However, the impact of FGS on progression-free survival and overall survival needs to be demonstrated in further prospective comparative trials (32). Consistent with the studies mentioned above, our data showed significantly higher GTR rates in the fluorescein group than in the non-fluorescein group (45.9% vs. 19.6%, P = 0.003; **Table 2**), and the PRTV was also significantly smaller in the fluorescein group than in the non-fluorescein group (0.40 [0.12-7.11) cm^3^ vs. 4.76 [0.44-11.00] cm^3^, P = 0.020, **Table 2**). However, our GTR rate, with or without the use of SF, was significantly lower compared to previous literature, which we speculate related to the following factors. First, the RANO criteria to defining GTR or PR are not frequently used in previous studies, e.g., Hong J et al. simply used postoperative cranial MRI-enhanced images and manual review to evaluate whether GTR was achieved and reported GTR rates of 85.7% (FG group) and 62.5% (NFG group) (7). Schebesch KM et al. used a blinded neuroradiologist for quantitative volume analysis and subsequently transferred the images to Brainlab iPlan cranial software (Brain Lab, Munich, Germany) for further evaluation of EOR, also achieving a satisfying GTR rate (40). Nevertheless, Hansen RW et al. using the RANO criteria for EOR determination, reported a GTR rate of only 62% for SF-guided HGG resection (4), which is consistent with our findings and again significantly below the range of GTR values reported in the majority of current literature. Second, some studies excluded data from patients with tumors located in the deep supratentorial region, such as Acerbi et al. (2013) (FLUOGLIO initial results) and Acerbi et al. (2018) (FLUOGLIO study). Data from patients with tumors located in the midline and basal ganglia regions were directly excluded from the above clinical trials (35, 42). Similarly, a retrospective study reported by Chen B et al. also excluded data from patients with tumor invading corpus callosum, midline, basal ganglia, etc (38). In contrast, our study included data from patients with tumors invading deep supratentorial regions (e.g., corpus callosum, basal ganglia, etc.), and the less favorable outcome of tumor resection in this group of patients decreased the GTR rate of the entire group due to the involvement of important neurological functional areas.

We further investigated the differences in GTR rates between the fluorescein and non-fluorescein groups according to different tumor locations in the supratentorial region. We found that in patients with tumors in the temporal and occipital lobes, the GTR rates in fluorescein patients were higher than in non-fluorescein patients. The PRTV was also significantly smaller in the fluorescein group than in the non-fluorescein group. In patients with tumors in the frontal and parietal lobes and the deep supratentorial region, the GTR rates and PRTV in both populations were not statistically significant, and further performed interaction and stratification analyses according to the adoption of SF-guided resection, EOR and tumor locations showing statistically significant differences. We speculate that the above mentioned findings may be related to the fact that the cerebral cortex’s language, motor, sensory, and cognitive areas are predominantly located in the frontal and parietal lobes and deep supratentorial region. The current surgical strategy for tumors affecting the cerebral cortex’s language, motor, sensory, and cognitive areas is to remove the tumor to the greatest extent possible safely; and to preserve vital neurological function as possible while extending patient survival to improve the postoperative quality of life, rather than pursuing total tumor resection based on neuroimaging (5, 43, 44). However, the tumor location classification method in this manuscript does not precisely distinguish between eloquent and non-eloquent tumors, and the above conclusions only suggest this potential possibility, which needs to be investigated by further prospective clinical trial data.

Current studies differ on the precise dose and timing of SF administration before surgery (4, 32, 33, 41, 45). Acerbi F et al. suggested the administration of a low dose (5 mg/kg) i.v. SF at the end of intubation (i.e. approximately 1 hour before dural opening), and this is currently the most commonly used procedure in HGG surgery under SF guidance (12, 42, 46). We followed and merged the procedure of Acerbi F and Schebesch et al, achieved satisfactory intraoperative tumor staining by intravenous injection of 0.5 mL SF for allergic testing before general anesthesia (to confirm the absence of allergic reactions). Subsequently, we administered a single intravenous injection of 3-5 mg/kg SF 20 min later if no allergic reaction was observed (7, 42, 46, 47). However, the extravasation and distribution of SF followed a specific time course. Intravascular fluorescein leaks out after a half-life of 264 min and may stain edema in the peritumoral normal brain parenchyma, increasing the risk of resectioning non-tumor tissue. Therefore, the timing of administration should be carefully planned to minimize these confounding factors (45). Hong et al. retrospectively analyzed 82 patients, 42 with FGS (fluorescein group) and 40 with NFGS (non-fluorescein group). They found that the fluorescein group experienced less intraoperative bleeding and shorter operating times than the non-fluorescein group (7). Our results also showed that the duration of surgery was significantly shorter in the fluorescein group than in the non-fluorescein group (P = 0.022; **Table 2**). However, the two groups had no statistically significant difference in intraoperative blood loss (P = 0.407, **Table 2**).

Further statistical analysis showed no statistically significant difference in intraoperative bleeding between tumors in different locations in the supratentorial region. In patients with frontal and occipital tumors, the duration of surgery was significantly shorter in the fluorescein group than in the non-fluorescein group. In contrast, there was no statistically significant difference between the two groups in patients with tumors in the temporal lobe, parietal lobe, and deep supratentorial region. Based on our previous surgical experience, we conclude that SF facilitates tumortumour tissue visualization and tumor margin delineation, theoretically reducing surgical bleeding and duration. However, an increase in the GTR rate also increases the workload of surgical resection and correspondingly prolongs the surgical duration. Surgical manipulation of brain tissue inherently disrupts the BBB, resulting in non-selective leakage of SF from the bloodstream along the cut edges of tumor tissue during surgery; this also affects the reliable delineation of tumor and normal tissue. In addition, the reduced illumination intensity in the fluorescence mode makes it more difficult to identify bleeding sites and stop bleeding in the surgical field. As a result, switching to the non-fluorescence mode is often necessary to achieve hemostasis, resulting in increased intraoperative bleeding and extended surgical duration. These effects are more pronounced in deeper tumor tissues. SF may stain the cerebrospinal fluid. Suppose the ventricular system is opened during resectioning of tumors in the ventricular system, and CSF flows into the surgical field. In that case, the field will appear yellow-green in fluorescence mode, making the procedure more difficult and requiring a switch back to non-fluorescence mode. However, this can also lead to increased intraoperative bleeding and prolonged surgical duration.

Our study also showed no statistically significant difference in the incidence of postoperative complications between patients with FGS and NFGS (P = 0.481, **Table 2**). The incidence of postoperative complications based on tumors in different locations in the supratentorial region was also not significantly different between the two groups. This result is similar to that reported by Hong et al. (7). In 2017, Acerbi et al. conducted a multicenter prospective phase II study of fluorescein sodium-guided high-grade glioma resection (FLUOGLIO), in which 40 serious adverse events occurred in 56 patients; none of the adverse events were considered related to SF administration (35). In 2022, Restelli F et al. reported an exceptional case of a young woman diagnosed with glioma and receiving a potentially toxic dose (almost 3 g) of SF during induction of anaesthesia due to a medical error. However, the surgical procedure was successfully completed with a Gross Total Tumor Resection and no clinical toxicities occurred. This case further reinforces the view that SF administration is a safe procedure (48). Consistent with the above-reported results, the current study also showed that fluorescein-guided glioma resection did not significantly increase the incidence of postoperative complications compared with conventional non-fluorescein procedures.

Several studies evaluated the effect of SF-guided glioma resection on survival: Koc et al. reported a median survival of 43.9 weeks for patients in the fluorescein group and 41.8 weeks for the non-fluorescein group. There was no statistically significant difference in survival between the two groups (49). Progression-free survival was significantly longer with fluorescein (7.2 months vs. 5.4 months; P = 0.033) in a study by Chen et al. However, this study lacked randomization and did not use special microscope filters to visualize fluorescence; only white light was used (38). Su X et al. performed a systematic computer search of PubMed and Web of Knowledge, including 12 studies, and a subsequent meta-analysis showed that SF-guided HGG resection was effective in reducing the risk of progression-free survival, with a pooled hazard ratio (HR) of 0.73 (95% CI 0.57-0.94, P = 0.01), but they also acknowledged that the above conclusions should be confirmed by subsequent randomized controlled clinical trials with large samples (50). In 2018, Acerbi F et al. reported a multicenter, prospective phase II clinical study on SF-guided resection of HGGs (FLUOGLIO) with a median follow-up of 11 months in 45 patients. PFS-6 and PFS-12 were 56.6% and 15.2%, respectively (PFS-6 and PFS-12 were defined as the proportion of patients who were progression-free at 6 and 12 months after diagnosis), and median survival was 12 months. However, they were unable to compare survival curves between the completely resected and incompletely resected subgroups due to the small number of incompletely resected cases (35). Schipmann S et al. summarized current strategies for intraoperative identification and targeting of glioblastoma and discussed the benefits and limitations of each technique in a 2019 review article. In their discussion of SF, the author stated that, to date, there are no reliable trial data on the impact of SF-guided resection on outcomes and survival (45), The review article summarised by Babu R et al. led to the same conclusion (51). Therefore, reliable data on the prognostic and survival impact of fluorescein-guided glioma resection have not been published (35, 45, 50, 51).

## 5 Conclusion

Fluorescein-guided resection of HGGs under a special operating microscope is feasible, safe, and convenient compared with conventional white light surgery without fluorescein guidance, significantly improves GTR rates, reduces PRTV, which is particularly beneficial for patients with tumors located in non-verbal, sensory, and motor and cognitive areas such as the temporal and occipital lobes, and does not increase the incidence of postoperative complications. However, because of the limited sample size of this study, this conclusion is preliminary. Further multicenter prospective studies with large samples are needed to demonstrate the prognostic impact of this technique.

## Data availability statement

The raw data supporting the conclusions of this article will be made available by the authors, without undue reservation.

## Ethics statement

The studies involving human participants were reviewed and approved by Ethics Committee of the First Affiliated Hospital of Xiamen University. Written informed consent to participate in this study was provided by the participants’ legal guardian/next of kin. Written informed consent was obtained from the individual(s) for the publication of any potentially identifiable images or data included in this article.

## Author contributions

CX: Conceptualization, methodology, writing- original draft preparation. SJ: Methodology, writing- original draft preparation. MJ: Methodology, writing-original draft preparation. CY: Data curation. LH: Data curation. SZ: Software. LZ: Validation. ZL: Formal analysis, Investigation. LY: Visualization. CS: Writing - review & editing. TG: Writing - review & editing, supervision. All authors contributed to the article and approved the submitted version.

## References

[1] Nayak L Reardon DA. High-grade gliomas. Continuum (Minneap Minn) (2017) 23(6):1548–63. doi: 10.1212/CON.0000000000000554 29200110

[2] de Groot JF. High-grade gliomas. Continuum (Minneap Minn) (2015) 21(2):332–44. doi: 10.1212/01.CON.0000464173.58262.d9 25837899

[3] Fathi Kazerooni A Bagley SJ Akbari H Saxena S Bagheri S Guo J. Applications of radiomics and radiogenomics in high-grade gliomas in the era of precision medicine. Cancers (Basel) (2021) 13(23):5921. doi: 10.3390/cancers13235921 34885031 PMC8656630

[4] Hansen RW Pedersen CB Halle B Korshoej AR Schulz MK Kristensen BW. Comparison of 5-aminolevulinic acid and sodium fluorescein for intraoperative tumor visualization in patients with high-grade gliomas: a single-center retrospective study. J Neurosurg (2019) 133:1–8. doi: 10.3171/2019.6.JNS191531 31585425

[5] Chen X Sun J Jiang W Zhu Z Chen S Tan G. Awake craniotomy for removal of gliomas in eloquent areas: an analysis of 21 cases. Brain Res Bull (2022) 181:30–5. doi: 10.1016/j.brainresbull.2021.12.017 34990734

[6] Schupper AJ Baron RB Cheung W Rodriguez J Kalkanis SN Chohan MO. 5-aminolevulinic acid for enhanced surgical visualization of high-grade gliomas: a prospective, multicenter study. J Neurosurg (2021) 136:1–10. doi: 10.3171/2021.5.JNS21310 34624862

[7] Hong J Chen B Yao X Yang Y. Outcome comparisons of high-grade glioma resection with or without fluorescein sodium-guidance. Curr Probl Cancer (2019) 43(3):236–44. doi: 10.1016/j.currproblcancer.2018.07.007 30119909

[8] Restelli F Mathis AM Höhne J Mazzapicchi E Acerbi F Pollo B. Confocal laser imaging in neurosurgery: a comprehensive review of sodium fluorescein-based CONVIVO preclinical and clinical applications. Front Oncol (2022) 12:998384. doi: 10.3389/fonc.2022.998384 36263218 PMC9574261

[9] Restelli F Pollo B Vetrano IG Cabras S Broggi M Schiariti M. Confocal laser microscopy in neurosurgery: state of the art of actual clinical applications. J Clin Med (2021) 10(9):2035. doi: 10.3390/jcm10092035 34068592 PMC8126060

[10] Orillac C Stummer W Orringer DA. Fluorescence guidance and intraoperative adjuvants to maximize extent of resection. Neurosurgery (2021) 89(5):727–36. doi: 10.1093/neuros/nyaa475 PMC8510852 33289518

[11] Manoharan R Parkinson J. Sodium fluorescein in brain tumor surgery: assessing relative fluorescence intensity at tumor margins. Asian J Neurosurg (2020) 15(1):88–93. doi: 10.4103/ajns.AJNS_221_19 32181179 PMC7057899

[12] Acerbi F Broggi M Eoli M Anghileri E Cavallo C Boffano C. Is fluorescein-guided technique able to help in resection of high-grade gliomas? Neurosurg Focus (2014) 36(2):E5. doi: 10.3171/2013.11.FOCUS13487 24484258

[13] Ung TH Kellner C Neira JA Wang SH D'Amico R Faust PL. The use of fluorescein sodium in the biopsy and gross-total resection of a tectal plate glioma. J Neurosurg Pediatr (2015) 16(6):732–5. doi: 10.3171/2015.5.PEDS15142 26407010

[14] Hamamcıoğlu MK Akçakaya MO Göker B Kasımcan MÖ Kırış T. The use of the YELLOW 560 nm surgical microscope filter for sodium fluorescein-guided resection of brain tumors: our preliminary results in a series of 28 patients. Clin Neurol Neurosurg (2016) 143:39–45. doi: 10.1016/j.clineuro.2016.02.006 26895208

[15] Waqas M Shamim MS. Sodium fluorescein guided resection of malignant glioma. J Pak Med Assoc (2018) 68(6):968–70. 30323373

[16] Catapano G Sgulò FG Seneca V Lepore G Columbano L di Nuzzo G. Fluorescein-guided surgery for high-grade glioma resection: an intraoperative "Contrast-enhancer". World Neurosurg (2017) 104:239–47. doi: 10.1016/j.wneu.2017.05.022 28512039

[17] Ahrens LC Krabbenhøft MG Hansen RW Mikic N Pedersen CB Poulsen FR. Effect of 5-aminolevulinic acid and sodium fluorescein on the extent of resection in high-grade gliomas and brain metastasis. Cancers (Basel) (2022) 14(3):617. doi: 10.3390/cancers14030617 35158885 PMC8833379

[18] Höhne J Schebesch KM de Laurentis C Akçakaya MO Pedersen CB Brawanski A. Fluorescein sodium in the surgical treatment of recurrent glioblastoma multiforme. World Neurosurg (2019) 125:e158–64. doi: 10.1016/j.wneu.2019.01.024 30682505

[19] Lim JX Loh D Tan L Lee L. Use of fluorescein sodium to obtain histological diagnosis of primary central nervous system lymphoma ghost tumour despite disappearance on intraoperative magnetic resonance imaging: technical note and review of the literature. Br J Neurosurg (2020), 1–5. doi: 10.1080/02688697.2020.1859087 33331187

[20] Okuda T Kataoka K Yabuuchi T Yugami H Kato A. Fluorescence-guided surgery of metastatic brain tumors using fluorescein sodium. J Clin Neurosci (2010) 17(1):118–21. doi: 10.1016/j.jocn.2009.06.033 19969462

[21] Schebesch KM Hoehne J Hohenberger C Acerbi F Broggi M Proescholdt M. Fluorescein sodium-guided surgery in cerebral lymphoma. Clin Neurol Neurosurg (2015) 139:125–8. doi: 10.1016/j.clineuro.2015.09.015 26432995

[22] Höhne J Brawanski A Schebesch KM. Fluorescence-guided surgery of brain abscesses. Clin Neurol Neurosurg (2017) 155:36–9. doi: 10.1016/j.clineuro.2017.02.014 28242559

[23] Save AV Gill BJ D’amico RS Canoll P Bruce JN. Fluorescein-guided resection of gliomas. J Neurosurg Sci (2019) 63(6):648–55. doi: 10.23736/S0390-5616.19.04738-6 31961117

[24] Ellingson BM Wen PY Cloughesy TF. Modified criteria for radiographic response assessment in glioblastoma clinical trials. Neurotherapeutics (2017) 14(2):307–20. doi: 10.1007/s13311-016-0507-6 PMC5398984 28108885

[25] Stummer W Pichlmeier U Meinel T Wiestler OD Zanella F Reulen HJ. Fluorescence-guided surgery with 5-aminolevulinic acid for resection of malignant glioma: a randomised controlled multicentre phase III trial. Lancet Oncol (2006) 7(5):392–401. doi: 10.1016/S1470-2045(06)70665-9 16648043

[26] Clavien PA Barkun J de Oliveira ML Vauthey JN Dindo D Schulick RD. The clavien-dindo classification of surgical complications: five-year experience. Ann Surg (2009) 250(2):187–96. doi: 10.1097/SLA.0b013e3181b13ca2 19638912

[27] Kommers I Ackermans L Ardon H van den Brink WA Bouwknegt W Balvers RK. Between-hospital variation in rates of complications and decline of patient performance after glioblastoma surgery in the dutch quality registry neuro surgery. J Neurooncol (2021) 152(2):289–98. doi: 10.1007/s11060-021-03697-8 PMC7997839 33511509

[28] Martin-Risco M Rodrigo-Paradells V Olivera-Gonzalez S Del Rio-Perez CM Bances-Florez L Calatayud-Perez JB. [Factors related with post-surgical complications in elderly patients with glioblastoma multiforme]. Rev Neurol (2017) 64(4):162–8. doi: 10.33588/rn.6404.2016246 28169411

[29] Wang LM Banu MA Canoll P Bruce JN. Rationale and clinical implications of fluorescein-guided supramarginal resection in newly diagnosed high-grade glioma. Front Oncol (2021) 11:666734. doi: 10.3389/fonc.2021.666734 34123831 PMC8187787

[30] Moore GE. Fluorescein as an agent in the differentiation of normal and malignant tissues. Science (1947) 106(2745):130–1. doi: 10.1126/science.106.2745.130-a 17750790

[31] Diaz RJ Dios RR Hattab EM Burrell K Rakopoulos P Sabha N. Study of the biodistribution of fluorescein in glioma-infiltrated mouse brain and histopathological correlation of intraoperative findings in high-grade gliomas resected under fluorescein fluorescence guidance. J Neurosurg (2015) 122(6):1360–9. doi: 10.3171/2015.2.JNS132507 25839919

[32] Acerbi F Cavallo C Broggi M Cordella R Anghileri E Eoli M. Fluorescein-guided surgery for malignant gliomas: a review. Neurosurg Rev (2014) 37(4):547–57. doi: 10.1007/s10143-014-0546-6 24756415

[33] Schebesch KM Brawanski A Hohenberger C Hohne J. Fluorescein sodium-guided surgery of malignant brain tumors: history, current concepts, and future project. Turk Neurosurg. (2016) 26:185–194. doi: 10.5137/1019-5149.JTN.16952-16.0 26956810

[34] Senders JT Muskens IS Schnoor R Karhade AV Cote DJ Smith TR. Agents for fluorescence-guided glioma surgery: a systematic review of preclinical and clinical results. Acta Neurochir (Wien) (2017) 159(1):151–67. doi: 10.1007/s00701-016-3028-5 PMC5177668 27878374

[35] Acerbi F Broggi M Schebesch KM Höhne J Cavallo C De Laurentis C. Fluorescein-guided surgery for resection of high-grade gliomas: a multicentric prospective phase II study (FLUOGLIO). Clin Cancer Res (2018) 24(1):52–61. doi: 10.1158/1078-0432.CCR-17-1184 29018053

[36] Sweeney JF Rosoklija G Sheldon BL Bondoc M Bandlamuri S Adamo MA. Comparison of sodium fluorescein and intraoperative ultrasonography in brain tumor resection. J Clin Neurosci (2022) 106:141–4. doi: 10.1016/j.jocn.2022.10.019 36327792

[37] Cooksey CJ. Quirks of dye nomenclature. 9. fluorescein. Biotech Histochem (2017) 92(7):506–12. doi: 10.1080/10520295.2017.1359751 28910172

[38] Chen B Wang H Ge P Zhao J Li W Gu H. Gross total resection of glioma with the intraoperative fluorescence-guidance of fluorescein sodium. Int J Med Sci (2012) 9(8):708–14. doi: 10.7150/ijms.4843 PMC3477680 23091408

[39] Kaneko S Eljamel MS. Fluorescence image-guided neurosurgery. Future Oncol (2017) 13(26):2341–8. doi: 10.2217/fon-2017-0194 29121788

[40] Schebesch KM Höhne J Rosengarth K Noeva E Schmidt NO Proescholdt M. Fluorescein-guided resection of newly diagnosed high-grade glioma: impact on extent of resection and outcome. Brain Spine (2022) 2:101690. doi: 10.1016/j.bas.2022.101690 36506293 PMC9729812

[41] Smith EJ Gohil K Thompson CM Naik A Hassaneen W. Fluorescein-guided resection of high grade gliomas: a meta-analysis. World Neurosurg (2021) 155:181–188.e7. doi: 10.1016/j.wneu.2021.08.126 34492388

[42] Acerbi F Broggi M Eoli M Anghileri E Cuppini L Pollo B. Fluorescein-guided surgery for grade IV gliomas with a dedicated filter on the surgical microscope: preliminary results in 12 cases. Acta Neurochir (Wien) (2013) 155(7):1277–86. doi: 10.1007/s00701-013-1734-9 23661063

[43] Lavrador JP Gioti I Hoppe S Jung J Patel S Gullan R. Altered motor excitability in patients with diffuse gliomas involving motor eloquent areas: the impact of tumor grading. Neurosurgery (2020) 88(1):183–92. doi: 10.1093/neuros/nyaa354 32888309

[44] Lavrador JP Gioti I Hoppe S Jung J Patel S Gullan R. In reply: altered motor excitability in patients with diffuse gliomas involving motor eloquent areas: the impact of tumor grading. Neurosurgery (2021) 88(3):E304–5. doi: 10.1093/neuros/nyaa514 33427292

[45] Schipmann S Schwake M Suero Molina E Stummer W. Markers for identifying and targeting glioblastoma cells during surgery. J Neurol Surg A Cent Eur Neurosurg (2019) 80(6):475–87. doi: 10.1055/s-0039-1692976 31466109

[46] Acerbi F Broggi M Broggi G Ferroli P. What is the best timing for fluorescein injection during surgical removal of high-grade gliomas? Acta Neurochir (Wien) (2015) 157(8):1377–8. doi: 10.1007/s00701-015-2455-z 26021579

[47] Schebesch KM Proescholdt M Höhne J Hohenberger C Hansen E Riemenschneider MJ. Sodium fluorescein-guided resection under the YELLOW 560 nm surgical microscope filter in malignant brain tumor surgery–a feasibility study. Acta Neurochir (Wien) (2013) 155(4):693–9. doi: 10.1007/s00701-013-1643-y 23430234

[48] Restelli F Bonomo G Monti E Broggi G Acerbi F Broggi M. Safeness of sodium fluorescein administration in neurosurgery: case-report of an erroneous very high-dose administration and review of the literature. Brain Spine (2022) 2:101703. doi: 10.1016/j.bas.2022.101703 36605385 PMC9808466

[49] Koc K Anik I Cabuk B Ceylan S. Fluorescein sodium-guided surgery in glioblastoma multiforme: a prospective evaluation. Br J Neurosurg (2008) 22(1):99–103. doi: 10.1080/02688690701765524 18224529

[50] Su X Huang QF Chen HL Chen J. Fluorescence-guided resection of high-grade gliomas: a systematic review and meta-analysis. Photodiagnosis Photodyn Ther (2014) 11(4):451–8. doi: 10.1016/j.pdpdt.2014.08.001 25131747

[51] Babu R Adamson DC. Fluorescence-guided malignant glioma resections. Curr Drug Discovery Technol (2012) 9(4):256–67. doi: 10.2174/157016312803305915 22339071

